# Supplementary material for: Observation of the spiral spin liquid in a triangular-lattice material
Source: Nat Commun. 2025 Mar 17;16:2619. doi: 10.1038/s41467-025-57319-2 (PMC11914452; doi:10.1038/s41467-025-57319-2)
Supplement: Supplementary file 1 — Supplementary Information [file 41467_2025_57319_MOESM1_ESM.pdf]

# Supplementary Information to accompany the article "Observation of the spiral spin liquid in a triangular-lattice material"

N. D. Andriushin, S. E. Nikitin, O. S. Fjellvag, J. S. White, A. Podlesnyak,
D. S. Inosov, M. C. Rahn, M. Schmidt, M. Baenitz, and A. S. Sukhanov

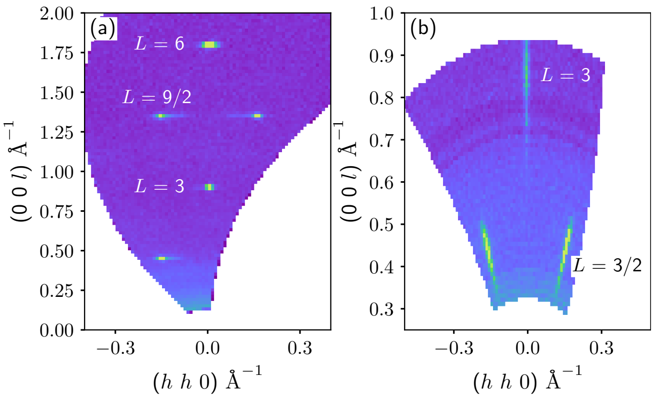


FIG. S1. (a) The scattering plane in the DMC experiment. (b) The scattering plane in the SANS-I experiment.

## S1. INSTRUMENTAL RESOLUTION IN EXPERIMENTAL DATA

### A. DMC experiment

The SSL state in $AgCrSe_2$ was confirmed by observation of a uniform ring in neutron diffraction data using the neutron diffractometer DMC and the neutron scattering instrument SANS-I. Before performing detailed analysis of the magnetic peaks, we first determined the instrumental resolution in both experimental setups, as this is required when magnetic-peak broadening is extracted. Because the resolution profile is strikingly different for DMC and SANS-I, the internal broadening can be reliably determined via cross analysis of the two datasets.

Because of high quality of the sample, the crystal-structure (nuclear) peaks were assumed resolution limited. Therefore, fits of the nuclear peaks allowed us to determine the instrumental resolution function. The observed broadening of the magnetic Bragg peaks can therefore be associated with intrinsic properties of the magnetic system.

As the magnetic reflections of interest are located close to $(0\,0\,4.5)$ r.l.u., we used the data on the nuclear Bragg peaks nearby: $(0\,0\,3)$ and $(0\,0\,6)$. The full $(H\,K\,4.5)$ maps of these peaks are shown in Figs. S2(a,b). The peak shape forms an ellipse elongated along one of the principal crystallographic directions. Therefore, we considered two perpendicular intensity profiles and fitted them with a Gaussian function, which yields the FWHM of the peak along the reciprocal $(h\,h\,0)$ and $(-k\,k\,0)$ directions [Figs. S2(c)–(f)]. Since the resolution depends on momentum transfer, the peak at (006) has larger FWHM, as can be seen from the corresponding profiles.

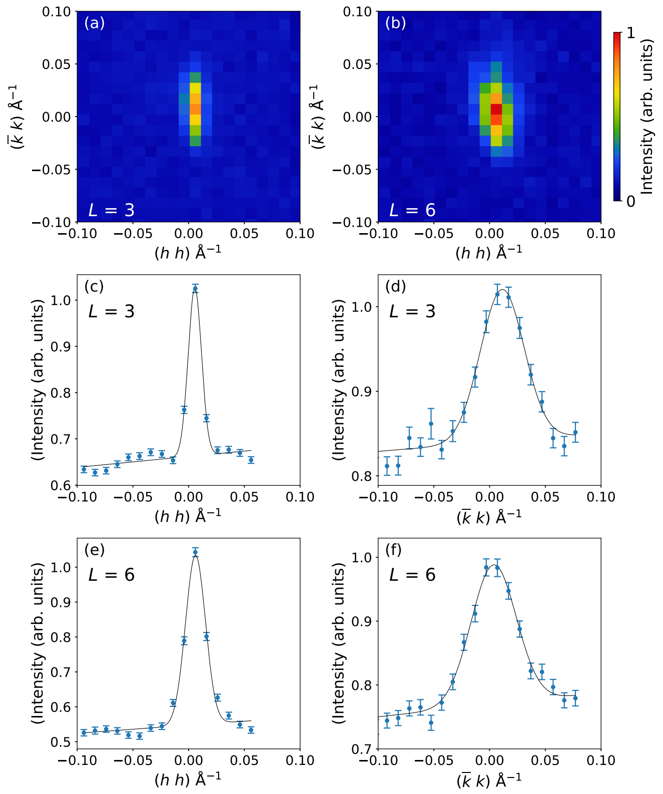


FIG. S2. The instrumental resolution in the DMC experiment determined by the nuclear reflections (003) and (006). (a,b) The intensity maps in the $(H\,K\,3)$ (a) and $(H\,K\,6)$ (b) reciprocal-space planes. (c,e) The intensity profiles of the (003) and (006) peaks along the $(h\,h\,0)$ direction, respectively. (d,f) The intensity profiles of the (003) and (006) peaks along the $(-k\,k\,0)$ direction, respectively.

The magnetic satellites $(\xi\,\xi\,4.5)$ are almost in between of these two structural reflections, meaning that the experimental resolution in the vicinity of $(0\,0\,4.5)$ can be well interpolated by an average of the two FWHM values of the $(0\,0\,3)$ and $(0\,0\,6)$ peaks. The interpolated values for the $(\xi\;\xi\;4.5)$ plane are then $0.018$ Å$^{-1}$ along $(h\,h\,0)$ and $0.047$ Å$^{-1}$ for $(-k\,k\,0)$.

For analysis of orientational disordering in $AgCrSe_2$, the diffraction pattern was rebinned into polar coordinates, allowing the extraction of azimuthal (in-plane angle) width to be done in a convenient way. For this purpose, the instrumental resolution can be directly recalculated as a function of azimuthal angle. For the zero azimuthal angle [the $(h\,h\,0)$ direction] and $|\mathbf{q}| = 0.15$ Å$^{-1}$, the instrumental angular FWHM $a = 16.7$ degrees. For the orthogonal direction [the $(-k\,k\,0)$ direction] it is determined to be $b = 6.5$ degrees. During the fitting, the angular resolution function at any par-

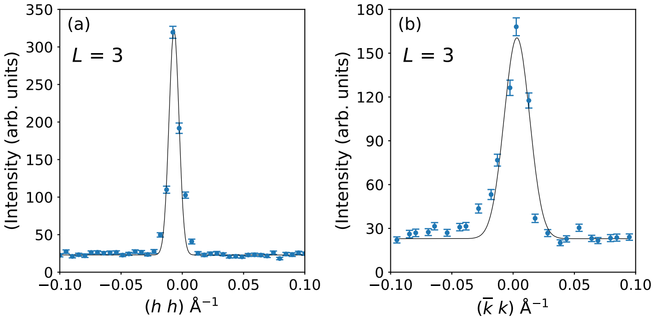


FIG. S3. The Gaussian fits to the nuclear (003) Bragg peak in the SANS-I experiment that show the instrumental resolution for the reciprocal $(hh0)$ (a) and $(-kk0)$ directions. The symbols are data, the solid lines are the fits.

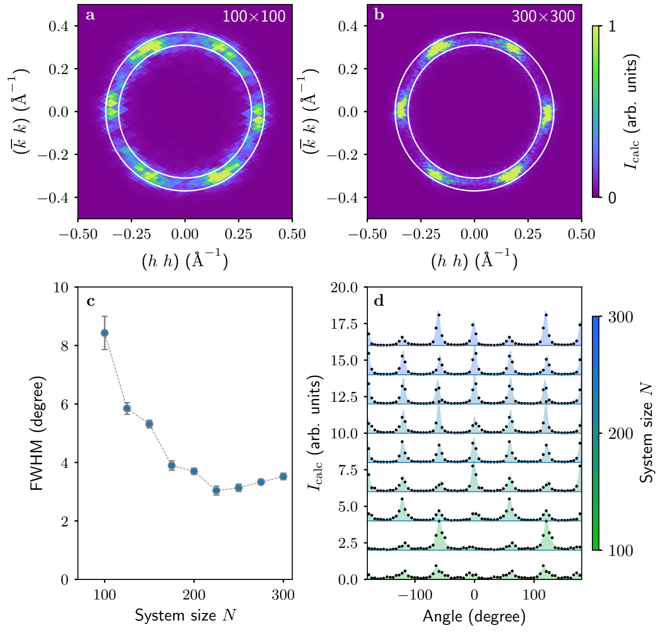


FIG. S4. The system-size tests. (a,b) Calculated structure factor for $100 \times 100$ and $300 \times 300$ systems. (c) Dependence of the azimuthal width on the system size. (d) Azimuthal dependence of the intensity obtained by an integration inside the area highlighted by the two white circles in (a) and (b). The color shading shows the Lorentzian fits, the curves were shifted by constant offsets for visual clarity.

ticular angle is assumed to be a Gaussian with the FWHM taken as interpolation between these two values: FWHM $= a\cos^2\phi + b\sin^2\phi$.

### B. SANS-I experiment

The momentum resolution profile of the SANS-I instrument has form distinct from that of DMC. Namely, the resolution ellipsoid is elongated radially in reciprocal space, i.e. along $(00L)$ for the nuclear (003) Bragg peak, as can be seen in the $(HHL)$ map in Fig. S1(b). The cross-section of the resolution ellipsoid in the perpendicular $(HK3)$ (crossing the nuclear peak) and $(H\,K\,3/2)$ (crossing the magnetic peaks) planes is also an ellipsoid for which the FWHM along $(h\,h\,0)$ and $(-k\,k\,0)$ directions differs by a factor of ∼2.5, which is similar to the DMC measurements. However, for both directions the FWHM is smaller than in the DMC setup, allowing for better refinement of the intrinsic SSL broadening. Figures S3(a) and S3(b) demonstrate Gaussian fits to the (003) peak along $(h\,h\,0)$ and $(-k\,k\,0)$ directions, respectively. The resulted instrumental resolution was then found as FWHM($hh0$) = 0.0096(3) Å$^{-1}$ and FWHM($-kk0$) = 0.0244(14) Å$^{-1}$. The FWHM in Å$^{-1}$ can again be recalculated into FWHM in degree for azimuthal cuts through the SSL state, which for the SANS-I measurements resulted in 1.8 and 4.6 degrees for $(h\,h\,0)$ and $(-k\,k\,0)$ directions respectively.

## S2. CALCULATION DETAILS

To reproduce the SSL state on the $J_1$-$J_2$-$J_3$ triangular lattice, we performed spin dynamics simulations using the Landau-Lifshitz dynamics approach as it implemented in the SU(N)NY program package [1]. The spins of Cr ions were represented as classical magnetic dipoles, and Heisenberg exchange interactions up to the third-nearest neighbor [as shown in Fig. 1(b) of the main text] were taken into account. The first-nearest neighbor exchange parameter $J_1$ was fixed to be ferromagnetic (FM), and all other exchange parameters are presented in units of $|J_1|$. In calculations, we assumed small easy $ab$-plane anisotropy, consistent with the previous reports [2]. We consider a single triangular lattice layer, as the interlayer interaction $J_c$ leads to only a trivial antiferromagnetic (AFM) coupling of the subsequent layers, which, in turn, responsible for the $L = 3/2$ r.l.u. component in the ordering vector $\mathbf{q}_{\rm m} = (0.045\ 0.045\ 3/2)$ r.l.u.. Moreover, the test calculations on the multilayered system showed that the combination of the FM $J_1$ and the AFM $J_c$ exchanges resulted only in the commensurate AFM magnetic order with the propagation vector $\mathbf{Q} = (0\,0\,3/2)$, indicating that the further-neighbor in-plane AFM exchange interactions are essential for the formation of the spiral order.

To achieve thermal equilibrium for the ground state and the structure factors calculations, a preliminary thermalization was performed. During the annealing process, the temperature was gradually lowered, while the spin configurations were sampled using Langevin dynamics [3]. To balance computational cost and accuracy, a system with periodic boundary conditions and the chosen size of $N \times N$ spins with $N = 300$.

Even though the exact parameters of magnetic interactions in $AgCrSe_2$ are currently unknown, it is still possible to achieve qualitative description of its magnetic properties. Focusing on the incommensurate phase III with ordering vector $\mathbf{q}_{\rm m} = (\xi\,\xi\,0)$, through the classical spin dynamics simulations we derive main general features attributed to $AgCrSe_2$.

The magnitude of the incommensurate propagation vector in the ground state depends on the values of $J_2$ and $J_3$ exchange interactions [see the phase diagram in Fig. 1(b) of the

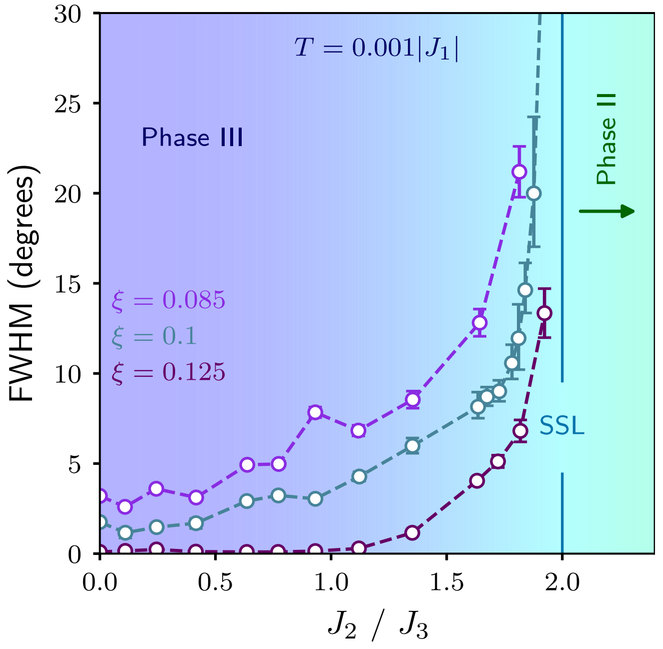


FIG. S5. Dependence of azimuthal width on the $J_2/J_3$ ratio for different magnitudes of the propagation vector.

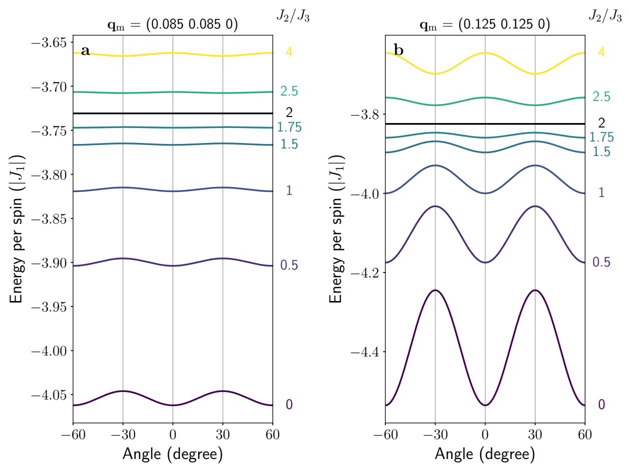


FIG. S6. Analytically calculated energy of a spin spiral as a function of the orientation of its propagation vector. Different curves correspond to different $J_2/J_3$ ratios. Zero angle is chosen as the (110) direction (the phase III) and the absolute value of the propagation $\mathbf{q}_\mathrm{m} = (0.085\,0.085\,0)$ for panel (a) and $\mathbf{q}_\mathrm{m} = (0.125\,0.125\,0)$ for panel (b).

main text]. For the phase III, the relation is given by [4]:

$$\xi = \arccos\Big(\frac{2J_2' - 2J_3' - \sqrt{(3J_2' + 2J_3')^2 + 8J_3'}}{-8J_3'}\Big), \quad \text{(S1)}$$

where $J_2' = J_2/J_1$ and $J_3' = J_3/J_1$. However, a smaller propagation vector magnitude, i.e. larger wavelength of spin modulation in real space, significantly increases computational expenses. Finite size effects become more prominent as the system contains fewer periods of modulated spins, necessitating a larger system size for better convergence and consequently more thermalization steps. Moreover, achieving thermal equilibrium for long-wave modulated spin textures requires more time steps due to the increased size of a magnetic unit cell. For these reasons, we consider a model system with exchange parameters that correspond to the propagation vector $\mathbf{Q} = (0.1\,0.1\,0)$, resulting in an approximately two times shorter wavelength of in-plane spin modulation as compared to the one observed in $AgCrSe_2$. This magnitude of the propagation vector imposes constraints on $J_2$ and $J_3$, which can be expressed by the following equation for phase III with $\mathbf{q}_\mathrm{m} = (\xi\,\xi\,0)$ as:

$$J_3 = \frac{-6\cos(2\pi\xi)J_2 + 3J_2 + 1}{\cos(2\pi\xi)[8\cos(2\pi\xi) - 4]}. \quad \text{(S2)}$$

This constraint sets the ratio $J_2/J_3$ as a free parameter that determines proximity of the system to the II–III critical phase boundary on the phase diagram [see Fig. 1(b) of the main text].

The magnetic order in phase III manifests as six peaks in the structure factor, corresponding to symmetry of the underlying crystal lattice. To analyze spin texture, we extract the azimuthal (in-plane angle) width of these peaks, which depends on the system's proximity to the transition from the phase III to the phase II controlled by the ratio $J_2/J_3$. By integrating the calculated structure factor radially, we obtain the azimuthal dependence of the intensity, which is fitted with a combination of Lorentzian peak functions with the identical full-width-half-maximum. An example of such fit is shown in Fig. S4(d). It is important to note that the calculated structure factor is discrete, therefore the quality of the rebinned data improves as larger systems have more Fourier modes present. During the fitting procedure, the peak centers are considered fixed, and the widths of all peaks from different domains are constrained to be equal.

In this type of calculations, a finite system size can naturally affect the simulation results. Therefore, it is essential to ensure that the degree of associated artifacts remains controllable. The system size effects were tested by checking the azimuthal width in relation to the number of spins in the system [Fig. S4(c)]. From the tests, we determine that the size less than $200 \times 200$ spins can introduce finite size effects that lead to enhanced peak width and large uncertainties in its determination. Note that the tests were carried out with exchange parameters corresponding to $\mathbf{q}_\mathrm{m} = (0.1\,0.1\,0)$, meaning that, for smaller propagation vectors, the larger system size and relaxation time are necessary in order to sustain this accuracy.

As mentioned earlier, the azimuthal width gradually increases as the exchange ratio $J_2/J_3$ approaches the value of 2.0, eventually becoming excessively broad. At this region, the width exceeds 60 degrees, compromising the reliability of the Lorentzian fit. This can be clearly seen on Fig. S5, where all data points in the figure calculated on the same convergence parameters and system size $300 \times 300$. The system was thermalized at fixed temperature $k_\mathrm{B}T = 0.001|J_1|$ after an-

nealing from $k_B T = 2|J_1|$. Since the chosen convergence parameters were optimized for $\xi = 0.1$, they become excessive for $\xi = 0.125$, resulting in decreased uncertainty. Oppositely, this produces a higher level of noise for $\xi = 0.085$. Nevertheless, it is clear that the azimuthal width is generally higher at a longer wavelength of the spin modulations.

The observed trend of the width response to the propagation vector length plausibly originates from the temperature condition in our simulations. To illustrate this, we analytically calculated the classical Heisenberg energy of a spiral state as a function of the spiral in-plane orientation angle. In Fig. S6, the zero angle was chosen along the (110) reciprocal-lattice direction [$\mathbf{q}_m = (0.085\,0.085\,0)$ r.l.u. in panel (a) and $\mathbf{q}_m = (0.125\,0.125\,0)$ r.l.u. in panel (b)]. As it can be seen, the energy has 60 degrees period of the in-plane orientation angle in accord to the underlying crystal-lattice symmetry. Changing frustration via the $J_2/J_3$ ratio, we can transit from the phase III (minimum energy is at $n \cdot 30°$ for $n$—even) into the phase II (minimum energy is at $n \cdot 30°$ for $n$—odd) through the SSL state with maximum frustration level of $J_2/J_3 = 2$, where the energy no longer depends on the propagation vector direction. It is important to note that all the features are preserved when the wavelength of the spiral is modified. However, the energy difference of the 30 and 0 degree spirals (i.e. the energy difference between the phase II and III) also depends on the total energy of the spiral, which, in turn, depends on the modulation wavelength. As was discussed earlier, in the simulations we extract the width of the peaks in the structure factor at a fixed temperature $k_B T = 0.001|J_1|$. In classical dipolar-spin simulations, the azimuthal width of the peaks is directly proportional to the softness of the spin spiral with respect to its in-plane orientation (the "transverse" stiffness). This energy dependence surely makes sense only in comparison with the temperature of a system. This means that the distribution of spiral orientation is different for a fixed temperature but different spiral period: shorter spiral would have more narrow profile as compared to long-period spiral due to reduced stiffens in the later case.

## S3. SIMULATIONS OF THE PANCAKE STATE AT HIGH TEMPERATURES

The structure factor of the paramagnetic state with correlations at finite propagation vector is demonstrated in Figs. S7 where it is shown for the parameters $J_2/J_3 = 0$ (no frustration) and $J_2/J_3 = 2$ (maximal frustration). As can be seen, this high-temperature state is characterized by a broad ring of intensity regardless of the frustration ratio. Unlike the SSL state, which has a well-defined static spin-spiral length, the correlated paramagnet in Figs. S7 represent spin fluctuations over a wide range of wavelengths.

At even higher temperatures, the broad ring gradually transforms into uncorrelated state [Fig. S8(a,b)]. An intermediate regime with finite residual correlations are referred in literature as "the pancake state" [5–7]. In our simulations, these correlations are generally weak at $T \gtrsim 3|J_1|$ and diminish to full extent at temperatures above $T \gtrsim 20|J_1|$ [Fig. S8(c)].

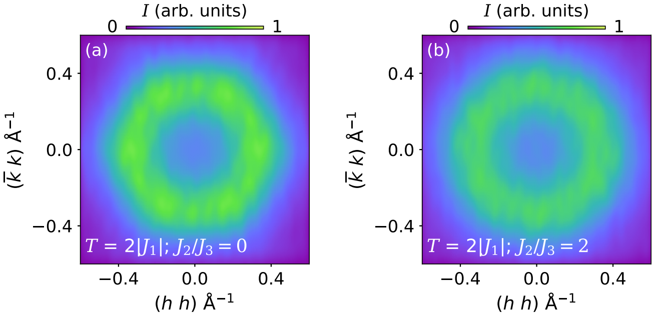


FIG. S7. The calculated structure factor in the paramagnetic state with correlations at finite momentum at $T = 2|J_1|$ for for $J_2/J_3 = 0$ (a) and $J_2/J_3 = 2$ (b). The convolution with the same experimental resolution was applied as in the main text.

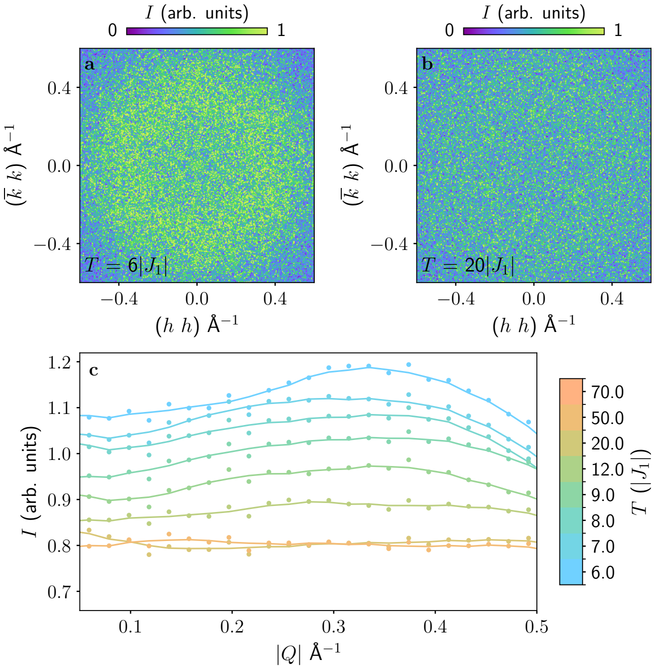


FIG. S8. The calculated structure factor at high temperature. (a,b) The structure factor at $T = 6|J_1|$ and $20|J_1|$, no convolution with experimental resolution applied. (c) The radial dependence of the intensity at elevated temperatures.

The same model parameters as in the main text were used for simulations shown in Fig. S8: $J_2 = 0.33|J_1|$, $J_3 = 0.19|J_1|$ and $K = 0.03|J_1|$.

## S4. HIGH TEMPERATURE DATA AND BACKGROUND SUBTRACTION IN THE SANS-I EXPERIMENT

At high temperatures, the SSL state expectedly looses its correlation length. At temperatures much greater than the crossover temperature, the spin spiral pitch is no longer preserved. As it fluctuates around a mean value, the ring-shaped structure factor broadens in the radial direction. Because

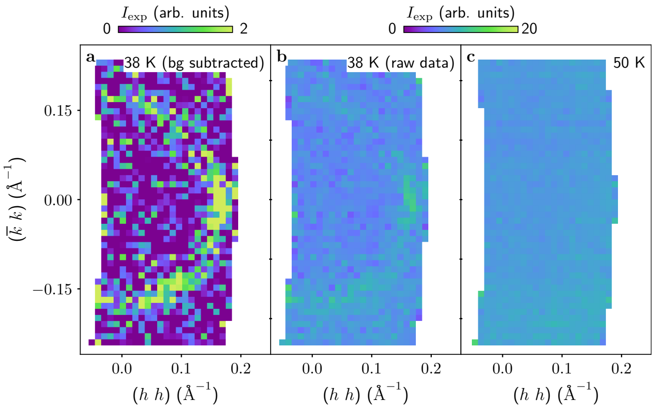


FIG. S9. The background subtraction in the SANS-I experiment. (a) Diffraction map at 38 K after background subtraction. (b) The same diffraction map at 38 K but before the background subtraction (raw data). (c) Diffraction map at 50 K, used as a background.

the SSL continuously transforms into the fully uncorrelated paramagnet over a wide temperature range, this intermediate partially-correlated state received a separate name "the pancake state" in the previous studies [5–7]. It is characterized by a broad structure factor, which describes both periodicity and orientation fluctuations of the spin spirals across the system. The structure factor eventually turns into a broad hump with the maxima at zero momentum at sufficiently high temperatures.

Since the intensity pattern of the pancake state is very broadened, it becomes very challenging to observe it in a neutron scattering experiment. The measurements are always accompanied by finite background signal coming from the incoherent scattering or scattering on the sample environment. Therefore, a weak scattering from the expected pancake state appears indistinguishable from the background level. Figure S9(c) shows the raw data collected at 50 K, where the inhomogeneous intensity (higher at the bottom) represent the background contained also in the measurements for all the other temperatures below 50 K. Because the pattern at 50 K is seemingly dominated by the background (the scattering from the pancake state is supposedly lower), we can use for background subtraction for all the measurements shown in the main text. Figures S9(a) and S9(b) demonstrate the SSL state at 38 K before and after the background subtraction. It can be seen, that the background-subtracted pattern allows for a more accurate analysis. Further experiments on significantly larger single crystals, which can improve the signal-to-noise ratio, are required for reliable observation of the weakly-correlated pancake state.

## S5. LOW-TEMPERATURE DEFECTS OF THE SSL

It should be noted, that at low temperatures, when the single ion magnetic anisotropy of $AgCrSe_2$ well overcomes its temperature fluctuations, the model used in our studies effectively turns into the XY model. The XY nature of the spins in $AgCrSe_2$ at low temperatures thus make it distinguished from the previously reported SSL materials such as $FeCl_3$ [8] and $MnSc_2S_4$ [9, 10] described by the Heisenberg 3D spins. Therefore, $AgCrSe_2$ represents a material for which the previous theoretical findings within the XY model of the SSL can be tested.

Yan and Reuther [6] considered the square lattice XY spin model of up to three near-neighbor exchange interactions. Similarly to the triangular-lattice model discussed in the main text, the square model of [6] predicts the SSL state for a certain combination of the frustrating exchange parameters. The Monte Carlo simulations performed by the authors of [6] showed that the stable spin configurations of the SSL may contain features that can be characterized as *momentum vortices*. The latter should not be confused with *the spin vortices*, such as the magnetic skyrmions and the magnetic bubbles, because the momentum vortices, as introduced in [6], are defined with respect to the vector field of the local directions of the spiral propagation vectors. In a full analogy with the spin vortices, the momentum vortices can have a winding number (the topological charge) attributed to them, which can take values $+1$ for the vortex, $-1$ for the antivortex, and 0 for a nontopological defect [6]. In configurations obtained with our Landau-Lifshitz dynamics calculations at a low temperature, similar momentum defects may occur.

Figures S10(a1) and S10(a2) demonstrate selected spots within a larger simulated system with two types of the defects found in our model of $AgCrSe_2$, which we label as the Defects I [Fig. S10(a1)] and the Defects II [Fig. S10(a2)]. To show that all the spin configurations within Fig. S10(a1) are equivalent but different from the spin textures of Fig. S10(a2) (which are also mutually equivalent within their set), we turn to the maps of the local propagation vectors where the momentum vortices can be identified. The momentum maps are calculated using equation

$$Q(\mathbf{r}) = \nabla \arctan(S_y/S_x), \tag{S3}$$

where $S_x$ and $S_y$ are the two Cartesian in-plane components of the spin.

As can be seen in Fig. S10(b1), each of the momentum map is characterized by a point where three different spiral regions, each with a well defined propagation vector direction $\sim$120 deg apart, join together. The three momenta around the joint form the "two-in, one-out" pattern, as schematically depicted in Fig. S10(c1). Whenever such a momentum junction is stabilized within the SSL state, the spin texture in the vicinity of it is found to feature a "bubble" (or a "loop") like the ones shown in Fig. S10(a1).

The spin textures reminiscent of the Y domain walls in Fig. S10(a2) appear as the result of the momentum vortices shown in Fig. S10(b2). There, a three momenta encircle the joint point in the fashion of Fig. S10(c2). As can be seen, the momentum vortex of the Defect II is rotation invariant to the Defect I. Namely, all the momenta are equally rotated by 90 deg.

We note that the Defect II structures are very similar to the configurations of winding number 0 predicted in [6]. Besides,

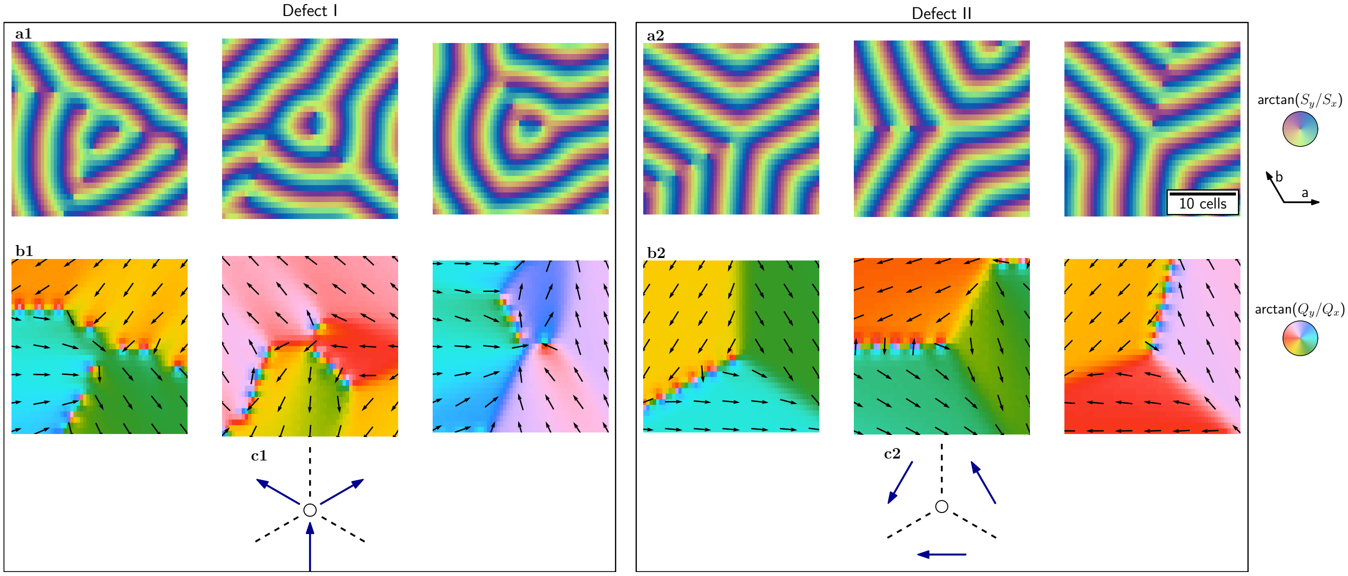


FIG. S10. Examples of the defects found in our simulations. Configurations were obtained after thermalization at $T = 0.0001|J_1|$ and the same model parameters as in the main text were used: $J_2 = 0.33|J_1|$, $J_3 = 0.19|J_1|$ and $K = 0.03|J_1|$. (a1,a2) Color-coded in-plane direction of the spins in the vicinity of the defects of type I and II. (b1,b2) The corresponding orientation of the spiral propagation vector in the vicinity of the defects of type I and II. (c1,c2) Schematics of the propagation vector junction of the defects of type I and II.

the authors of [6] predicted the topological configurations of winding numbers $+1$ and $-1$, which represent the "all-in" (contrary to "two-in, one-out") momentum pattern around the joint point. Such defects were not observed in our simulations.


[1] H. Zhang and C. D. Batista, Classical spin dynamics based on SU($N$) coherent states, Phys. Rev. B **104**, 104409 (2021).

[2] M. Baenitz, M. M. Piva, S. Luther, J. Sichelschmidt, K. M. Ranjith, H. Dawczak-Debicki, M. O. Ajeesh, S.-J. Kim, G. Siemann, C. Bigi, P. Manuel, D. Khalyavin, D. A. Sokolov, P. Mokhtari, H. Zhang, H. Yasuoka, P. D. C. King, G. Vinai, V. Polewczyk, P. Torelli, J. Wosnitza, U. Burkhardt, B. Schmidt, H. Rosner, S. Wirth, H. Kühne, M. Nicklas, and M. Schmidt, Planar triangular $AgCrSe_2$: Magnetic frustration, short range correlations, and field-tuned anisotropic cycloidal magnetic order, Phys. Rev. B **104**, 134410 (2021).

[3] D. Dahlbom, C. Miles, H. Zhang, C. D. Batista, and K. Barros, Langevin dynamics of generalized spins as SU($N$) coherent states, Phys. Rev. B **106**, 235154 (2022).

[4] C. Glittum and O. F. Syljuåsen, Arc-shaped structure factor in the $J_1$-$J_2$-$J_3$ classical heisenberg model on the triangular lattice, Phys. Rev. B **104**, 184427 (2021).

[5] T. Shimokawa and H. Kawamura, Ripple state in the frustrated honeycomb-lattice antiferromagnet, Phys. Rev. Lett. **123**, 057202 (2019).

[6] H. Yan and J. Reuther, Low-energy structure of spiral spin liquids, Phys. Rev. Res. **4**, 023175 (2022).

[7] M. G. Gonzalez, A. Fancelli, H. Yan, and J. Reuther, Magnetic properties of the spiral spin liquid and surrounding phases in the square lattice XY model, Phys. Rev. B **110**, 085106 (2024).

[8] S. Gao, M. A. McGuire, Y. Liu, D. L. Abernathy, C. dela Cruz, M. Frontzek, M. B. Stone, and A. D. Christianson, Spiral Spin Liquid on a Honeycomb Lattice, Phys. Rev. Lett. **128**, 227201 (2022).

[9] S. Gao, O. Zaharko, V. Tsurkan, Y. Su, J. S. White, G. S. Tucker, B. Roessli, F. Bourdarot, R. Sibille, D. Chernyshov, T. Fennell, A. Loidl, and C. Rüegg, Spiral spin-liquid and the emergence of a vortex-like state in $MnSc_2S_4$, Nature Phys. **13**, 157 (2016).

[10] S. Gao, H. D. Rosales, F. A. G. Albarracín, V. Tsurkan, G. Kaur, T. Fennell, P. Steffens, M. Boehm, P. Čermák, A. Schneidewind, E. Ressouche, D. C. Cabra, C. Rüegg, and O. Zaharko, Fractional antiferromagnetic skyrmion lattice induced by anisotropic couplings, Nature **586**, 37 (2020).
